# Correspondence

**Published:** 1883-06

**Authors:** X. C. Scott

**Affiliations:** Chairman Com. of Arrangements; Cleveland, O.


THE following letter has been received from X. C. Scott, M.D., Chairman of the Committee of Arrangements for the meeting in Cleveland, Ohio, of the American Medical Association.

CLEVELAND, O., May 8th, 1883.

DEAR SIR—The next annual meeting of the American Medical Association will be held in this city June 5th to 8th, inclusive. All railroads west of Pittsburg, Salamanca and Buffalo, east of Chicago and south of Cleveland, will carry delegates and members of their families to Cleveland at one full fare, and return them on certificate signed by me as Chairman of the Committee of Arrangements (certifying that they have been in attendance at the meetings of the Association) at one cent per mile.

The Trunk Line east of Buffalo, Salamanca and Pittsburg, and lines west of Chicago, have refused to make any reduction.

The rates per diem at the hotels will be: Kennard House, $3.00; Meddell House, $3.00; Forest City House, $2 50 to $3.00; American House, $2.50; Hawley House, $2.00; Streibinger House, $2.00; Clarendon House, $2.00, Prospect House, $2.00.

Please make notice of this in your next issue for the benefit of the profession.

Truly yours, X. C. SCOTT,
*Chairman Com. of Arrangements.*

